# Expression Of Intracellular Components of the NF-κB Alternative Pathway (NF-κB2, RelB, NIK and Bcl3) is Associated With Clinical Outcome of NSCLC Patients

**DOI:** 10.1038/s41598-019-50528-y

**Published:** 2019-10-04

**Authors:** Foteinos-Ioannis D. Dimitrakopoulos, Anna G. Antonacopoulou, Anastasia E. Kottorou, Nikolaos Panagopoulos, Fotini Kalofonou, Fotios Sampsonas, Chrisoula Scopa, Melpomeni Kalofonou, Angelos Koutras, Thomas Makatsoris, Dimitrios Dougenis, Helen Papadaki, Malcolm Brock, Haralabos P. Kalofonos

**Affiliations:** 1 Molecular Oncology Laboratory, Division of Oncology, Department of Internal Medicine, Medical School, University of Patras, Patras, Greece; 2 Department of Cardiothoracic Surgery, Medical School, University of Patras, Patras, Greece; 3 Department of Respiratory Medicine, University Hospital of Patras, Patras, Greece; 4 Department of Pathology, Medical School, University of Patras, Patras, Greece; 5 Institute of Biomedical Engineering, Imperial College London, London, United Kingdom; 6 Department of Anatomy, Medical School, University of Patras, Patras, Greece; 7 Division of Thoracic Surgery, Department of Surgery, School of Medicine, Johns Hopkins University, Baltimore, MD USA

**Keywords:** Lung cancer, Non-small-cell lung cancer

## Abstract

A growing number of studies has shed light on the role of the NF-κΒ in non-small-cell lung cancer (NSCLC). To address the significance of major effectors of the NF-κΒ alternative pathway, we investigated the relationship between NF-κΒ2, RelB, NIK and Bcl3 expression (mRNA and protein) and the clinical outcome of NSCLC patients. NF-κΒ2, RelB, NIK and Bcl3 protein expression levels were assessed by immunohistochemistry in tissue samples from 151 NSCLC patients who had curative resection. mRNA levels were also evaluated in 69 patients using quantitative real-time PCR. Although all studied proteins were overexpressed in NSCLC (P < 0.001 for all), only *RelB* mRNA levels were strongly increased in cancerous specimens compared to tumor-adjacent non-neoplastic tissues (P = 0.009). Moreover, NF-κB2, RelB and Bcl3 expression was associated with overall survival (OS). In particular, cytoplasmic and mRNA expression of RelB was related to 5-year OS (P = 0.014 and P = 0.006, respectively). Multivariate analysis also showed that Bcl3 expression (nuclear and cytoplasmic) was associated with increased 5-year OS (P = 0.002 and P = 0.036, respectively). In addition, higher *Bcl3* mRNA levels were associated with inferior OS in stages I & II and improved OS in stages III and IV after 5-year follow-up (P = 0.004 and P = 0.001, respectively). Furthermore, stage I patients with lower *NF-κB2* mRNA levels had better 5-year survival in univariate and multivariate analysis (P = 0.031 and P = 0.028, respectively). Interestingly, RelB expression (cytoplasmic and mRNA) was inversely associated with relapse rates (P = 0.027 and P = 0.015, respectively), while low NIK cytoplasmic expression was associated with lower relapse rates (P = 0.019). Cytoplasmic NIK expression as well as NF-κB2/ Bcl3 detection was associated with lymph node infiltration (P = 0.039 and P = 0.014, respectively). The present study confirms the deregulation of the NF-κB alternative pathway in NSCLC and also demonstrates the importance of this pathway in prognosis, recurrence and infiltration of regional lymph nodes.

## Introduction

Lung cancer is the leading cause of cancer-related deaths worldwide in both sexes with more than 1.8 million deaths per year in males^1^. Anti-smoking policies in some countries during the last decades have led to a decrease in the prevalence of the disease^2^. However, global future projections are rather worrying, due to, on the one hand, the lack of consistency of anti-smoking policies in the majority of countries and, on the other hand, the predicted increase of exposure to known carcinogens (smoke, air pollution etc), especially in developing countries^3,4^.

Non-small-cell lung cancer (NSCLC) remains the major histological subtype of lung cancer (80–85% of cases), with small-cell lung cancer accounting for 15% of lung cancer patients^5^. Recent advances in targeted drugs and immunotherapeutic interventions (checkpoint inhibitors) have improved treatment response rates in specific patient subgroups, but even more effective therapies are needed to achieve a significant improvement in the survival outcome of lung cancer patients^6^. A better understanding of the tumor’s molecular characteristics and a faster translation of our current knowledge into clinically useful tools remains an unmet need for patients and the scientific community alike^7^.

Analysis of signaling pathways in lung cancer pathogenesis conducted over the years offers insight into the pathogenesis and the progression of this disease. The nuclear factor kappa-light-chain-enhancer of activated B cells (NF-κB) transcription factor pathway has been one of the key pathways studied, but has been characterized as a “double-edged sword” due to its pivotal role in the promotion of inflammation and tumor development, as well as in the regulation of the immune system against cancer^8^. In addition, this pathway plays an important role in the development of different types of immune cells (B cell and lymphoid organogenesis), as well as hematopoietic stem cells (HSC), while its deregulation has been documented in rheumatologic diseases and in cancer^9–12^.

**Figure 1:**
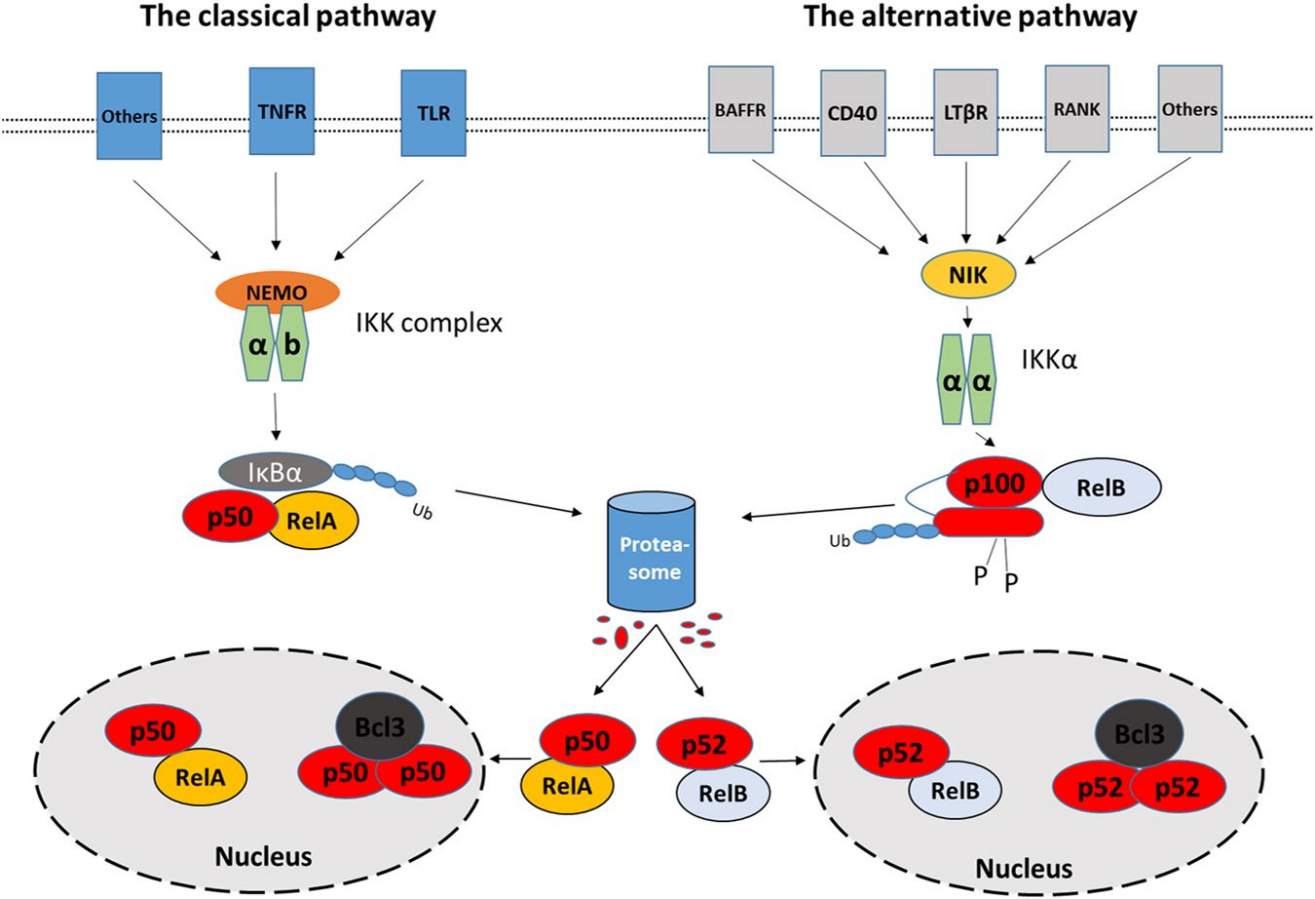
The two major NF-κB pathways (classical and the alternative). The activation of the classical pathway mainly leads to the formation of an active heterodimer of p50:RelA, which modifies multiple gene expression by binding to κB binding sites. The alternative pathway regulates gene expression through the binding of the central complex p52:RelB. Many other heterodimers and homodimers of p50 and p52 are formed increasing further the complexity of the NF-κB system. Abbreviations: TLR; Toll-like receptors, TNFR; Tumor necrosis factor receptor, NEMO; NF-kappa-B essential modulator, IκB; nuclear factor of kappa light polypeptide gene enhancer in B-cells inhibitor, BAFFR; tumor necrosis factor receptor superfamily member 13 C, CD40; CD40 molecule, TNF receptor superfamily member 5, LTβR; Lymphotoxin Beta Receptor (TNFR Superfamily, Member 3), RANK; Receptor Activator Of Nuclear Factor-Kappa B, NIK; NF-Kappa-Beta-Inducing Kinase, IKKα; IκB Kinase α, IKKb; IκB Kinase b, p100; nuclear factor NF-kappa-B p100 subunit, p52; nuclear factor NF-kappa-B p52 subunit, RelB; Transcription factor RelB, Bcl3; B-Cell CLL/Lymphoma 3.

The seven members [p105/p50 (NF-κB1), p100/p52 (NF-κB2), p65 (RelA), RelB, c-Rel] of the NF-κB family are encoded by five genes (*NF-κB1*, *NF-κB2*, *RELA*, *RELB*, *c-REL*)^13,14^. *NF-κB1* and *NF-κB2* genes are responsible for the transcription of p105 and p100 proteins, respectively, which in turn are cleaved by proteasomes leading to the functional molecules p50 and p52, respectively^14,15^. The seven effector molecules of the family exert their function through the activation of two pathways, which are termed “classical” and “alternative”^16^. The central players of the classical pathway are the p65 and p50 subunits while in the alternative pathway the central transcriptionally active heterodimer is the p100/p52:RelB complex (Fig. 1).

Only in the last few years has the less well-known alternative pathway of NF-κB attracted the interest of the scientific community with an increasing amount of data implicating this pathway to NSCLC pathogenesis. Our group was the first to demonstrate that at the protein level the two central components of the alternative pathway, p100/p52 and RelB, were overexpressed in primary NSCLC lesions compared to adjacent non-neoplastic lung parenchyma and normal, cadaveric lung tissues in individuals who have never been exposed to cigarette smoke^17^. Additionally, we showed that BCL3 protein expression was also elevated in NSCLC^18^. Recently, Saxon *et al*. demonstrated in a transgenic mouse model that overexpression of p52 in airway epithelial cells after lipopolysaccharide (LPS) stimulation leads to reduced cellular survival and increased expression of several pro-apoptotic genes^19^. The purpose of this study was to evaluate in a comprehensive way the expression of the major intracellular players of the NF-κB alternative pathway of (NF-κΒ2, RelB, NIK and Bcl3) at the protein and mRNA levels, and to investigate the significance of these molecules as prognostic factors in NSCLC patients.

## Results

### Clinical and pathological characteristics of the patient cohort

The clinicopathological characteristics of the study population are summarized in Supplementary Table S1. The median age of the patients was 66 years, with a range of 40 to 84 years. Primary pathology reports were used to define the pathological stage. Samples from stages I to III were included and were equally distributed. Eighty-six samples were squamous cell carcinomas, 54 were adenocarcinomas and 10 were large cell carcinomas. Nodal metastatic status was known for 144 patients, of which 52.3% were found to have infiltration of the regional lymph nodes. The second, third, and fifth year survival outcomes were available in 149, 146 and 146 patients, respectively. Moreover, the regional relapse status after 2-year follow-up was known for 37 patients (23 relapsed).

### NF-κB2, RelB, NIK, Bcl3 are overexpressed in NSCLC

**Figure 2:**
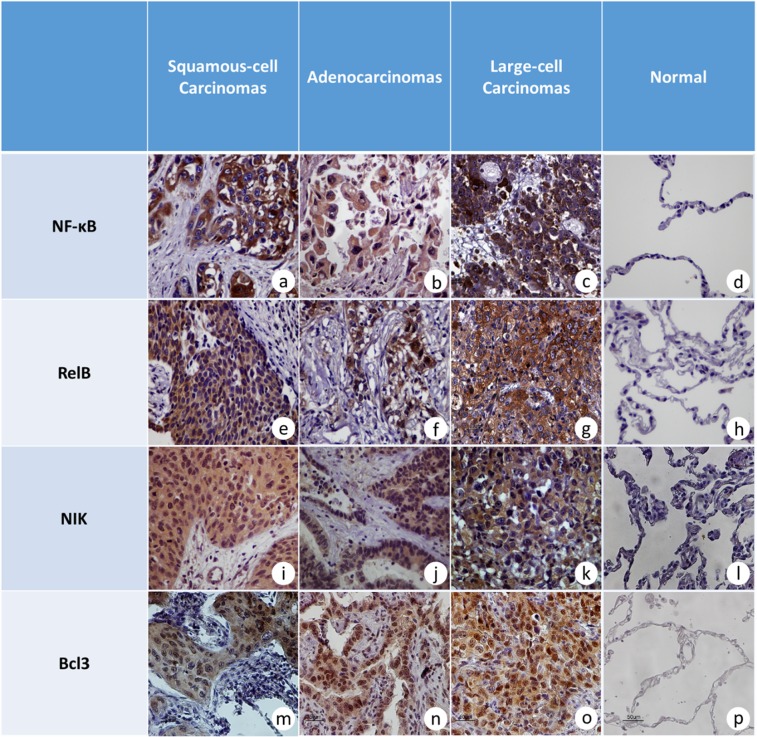
Microphotographs (×40) from tumor and tumor-adjacent, non-cancerous samples. (**a**) NF-κB2 in a grade II, squamous lung carcinoma with strong and intermediate cytoplasmic staining without nuclear signal, (**b**) NF-κB2 intermediate cytoplasmic staining in adenocarcinoma, (**c**) strong and intermediate cytoplasmic signal for NF-κB2 in an undifferentiated large-cell carcinoma, (**d**) negative immunostaining for NF-κB2 protein in alveolar epithelium and interstitium of tumor-adjacent non-neoplastic lung parenchyma, (**e**) RelB immunodetection in a grade III, squamous-cell carcinoma with strong cytoplasmic staining, (**f**) strong nuclear and cytoplasmic staining for RelB protein in grade III lung adenocarcinoma, (**g**) representative section of undifferentiated large-cell, lung carcinoma with strong cytoplasmic signal for RelB, (**h**) negative immunostaining for RelB in alveolar epithelium and interstitium of tumor-adjacent non-neoplastic lung parenchyma from a squamous-cell carcinoma, (**i**) representative section from a grade I, squamous -cell carcinoma with strong cytoplasmic staining for NIK, (**j**) intermediate cytoplasmic staining for NIK in an adenocarcinoma, (**k**) strong cytoplasmic immunostaining for NIK in large-cell lung carcinoma, (**l**) negative immunostaining for ΝΙΚ in alveolar epithelium and interstitium of adjacent non-neoplastic lung parenchyma from a large-cell carcinoma, (**m**) representative section of grade I, squamous-cell carcinoma with strong cytoplasmic and intermediate nuclear staining for Bcl3, (**n**) strong cytoplasmic staining for Bcl3 in a lung adenocarcinoma, (**o**) intermediate nuclear and strong cytoplasmic staining for Bcl3 protein in a large-cell lung carcinoma, (**p**) negative immunostaining for Bcl3 in alveolar epithelium of tumor-adjacent, non-neoplastic lung parenchyma.

**Figure 3:**
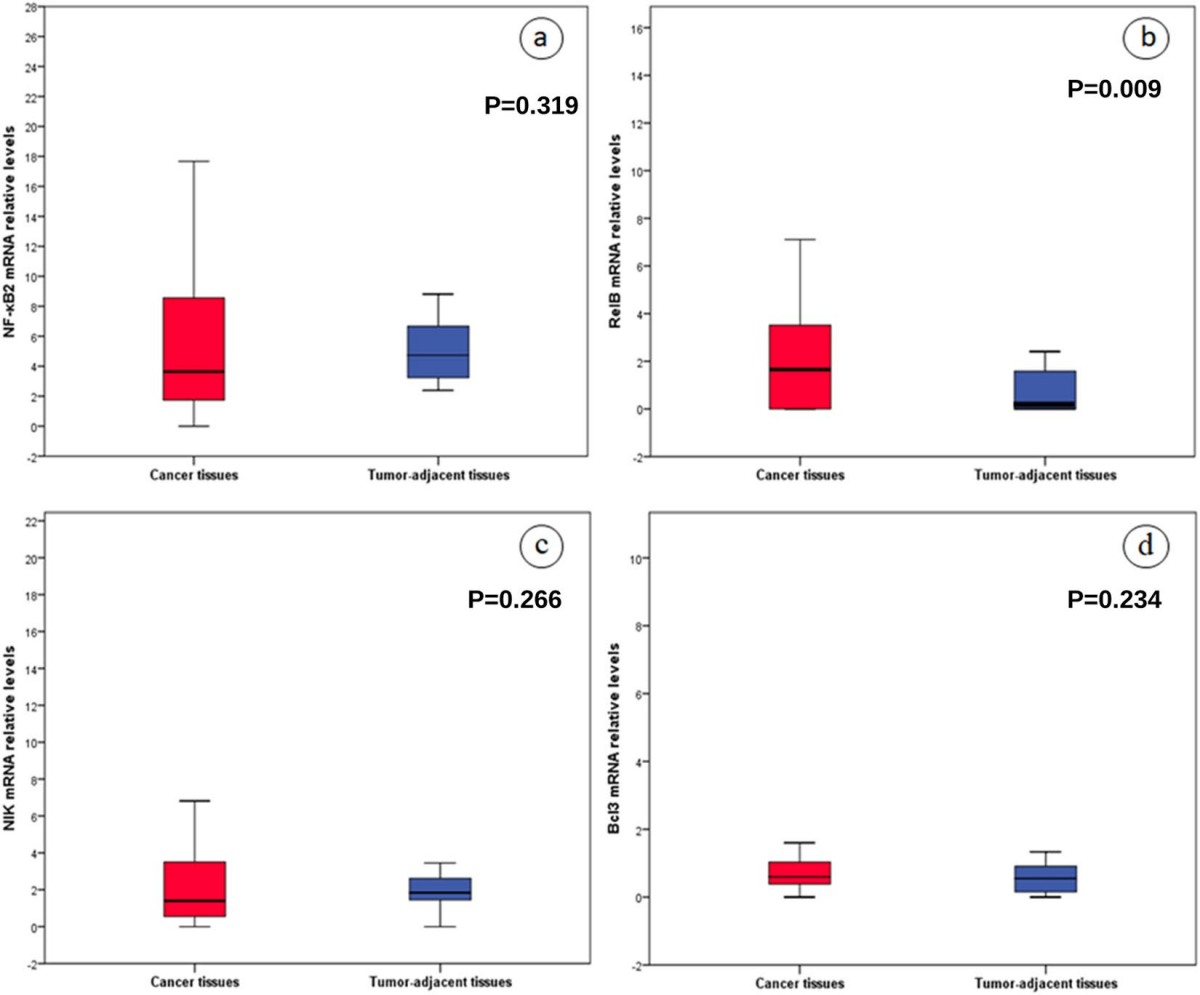
Relative expression in mRNA levels of (**a**) *NF-κB2*, (**b**) *RelB*, (**c**) *NIK* and (**d**) *Bcl3* genes in tumor and tumor-adjacent specimens.

All studied molecules were expressed more frequently in tumor tissues compared to tumor-adjacent, non-neoplastic tissues. In particular, cytoplasmic NF-κB2 and RelB were detected in 97.4% and 67.1% of NSCLC specimens, while nuclear immunostaining for NF-κB2 and RelB was observed in 18.7% and 47.2%, respectively. On the contrary, NF-κB2 and RelB were observed in both compartments in only 10% of tumor-adjacent non-neoplastic tissues (Fig. 2). Moreover, expression levels of NF-κB2 and RelB were significantly higher in tumors compared to non-neoplastic tissues (Fig. 2, P < 0.001 for both). Bcl3 and NIK were also detected in most NSCLC cases (100% and 92.5%, respectively), while no signal was detected in adjacent non-neoplastic specimens (Fig. 2, P < 0.001 for both of them). With regard to mRNA expression, RelB mRNA levels were strongly increased in cancerous specimens compared to tumor-adjacent non-neoplastic tissues (Fig. 3b, P = 0.009), while mRNA levels of NF-κB2, Bcl3 and NIK did not differ between neoplastic and non-neoplastic tissues (Fig. 3).

### Expression levels of RelB, Bcl3, ΝΙΚ and NF-κB2 were associated with overall survival

**Figure 4:**
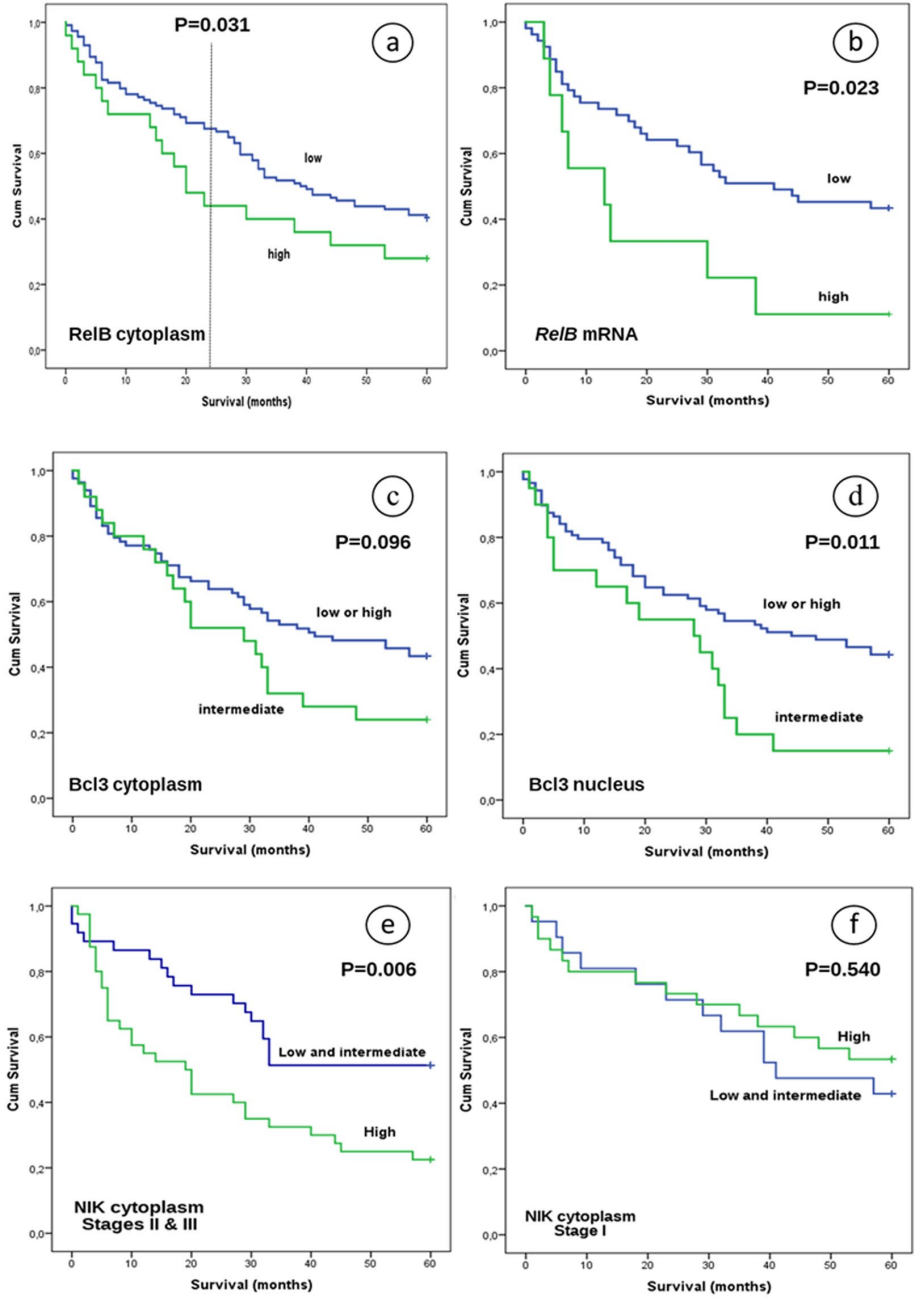
Overall survival of NSCLC patients after 5-year follow-up with regard to (**a**) RelB cytoplasmic signal, (**b**) *RelB* mRNA expression, (**c**) Bcl3 cytoplasmic protein expression, (**d**) Bcl3 nuclear protein expression, (**e**) NIK cytoplasmic expression of stages II and III patients and (**f**) NIK cytoplasmic expression of stage I patients.

By univariate analysis, patients with low or intermediate cytoplasmic expression of RelB had improved 2-year survival compared to patients with higher expression levels (P = 0.031). However, this difference was lost when assessing 3- and 5-year survival (Fig. 4a, P = 0.183 and P = 0.128, respectively). Survival was also associated with *RelB* mRNA levels (Fig. 4b, P = 0.023 for 5-year follow-up). In addition, the prognostic significance of the cytoplasmic and mRNA expression of RelB for 5-year OS was also observed using multivariate Cox proportional hazards models adjusted for age, grade, primary location, smoking, stage, histological subtype and maximum diameter (P = 0.014; HR, 0.288; 95% CI, 0.107–0.776 and P = 0.006; HR, 1.242; 95% CI, 1.065–1.449, respectively).

**Figure 5:**
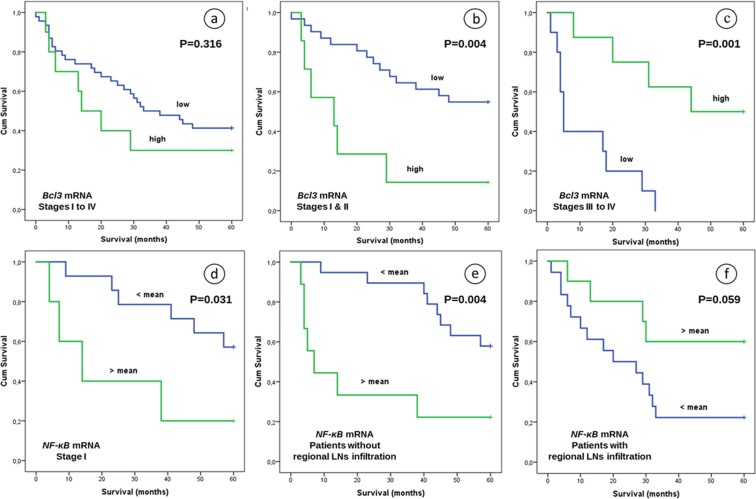
Overall survival of NSCLC patients after 5 years observation in relation to: (**a**) *Bcl3* mRNA expression in patients of stages I to IV (cut-off point 2.09), (**b**) *Bcl3* mRNA expression in patients of stages I and II (cut-off point 2.09), (**c**) *Bcl3* mRNA expression in stages III & IV (cut-off point 0.43), (**d**) *NF-κΒ2* mRNA expression in stage I patients, (**e**) *NF-κΒ2* mRNA expression in patients without regional lymph node (LN) infiltration and (**f**) *NF-κΒ2* mRNA expression in patients with regional LN infiltration. P values refer to univariate analysis.

With regard to Bcl3, both protein and mRNA expression levels were associated with OS. Low or high nuclear or cytoplasmic protein levels were associated with worse 5-year survival outcome compared to intermediate expression in univariate analysis (Fig. 4c,d, P = 0.011 and P = 0.096, respectively). Furthermore, these observations were statistically significant in Cox regression models, using the age, grade, gender, histological subtype and primary location as coefficients (P = 0.002; HR, 2.588; 95% CI, 1.409–4.751 and P = 0.036; HR, 1.901; 95% CI, 1.044–3.460, respectively). Although mRNA levels appeared to have a poor prognostic value in the cohort as a whole (Fig. 5a, P = 0.316), stratification according to stage and using a cut-off point of 2.09, showed an inferior OS in stage I and II patients with high *Bcl3* mRNA levels (Fig. 5b) not only in univariate (P = 0.004), but also in multivariate analysis (age, grade, histological subtype, primary location, maximum diameter, smoking as coefficients, P = 0.030). Higher *Bcl3* mRNA levels in stages III and IV were associated with improved clinical outcome after a period of 5-year observation, which was statistically significant using a cut-off point of 0.43 as determined by the X-tile tool in this subpopulation (Fig. 5c, P = 0.001). In addition, this association remained after multivariate analysis (P = 0.013; HR, 0.493; 95% CI, 0.283–0.859).

Interesting was also the observation that although NIK cytoplasmic and mRNA levels weren’t associated with OS in the whole cohort (P = 0.125 and P = 0.760, respectively), further stratification based on disease stage showed a significant association of NIK cytoplasmic signal with OS. Particularly, patients of stages II and III with low or intermediate levels had better clinical outcome after 5-year observation (P = 0.006, Fig. 4e), but not patients of stage I (P = 0.540, Fig. 4f). This correlation continued to be statistically significant in Cox regression models, using age, grade, gender, histological subtype and primary location as coefficients (P = 0.035; HR, 0.475; 95% CI, 0.237–0.951).

Another interesting finding was the association of the *NF-κB2* mRNA expression with OS after stratification with pathological stage. In particular, patients of stage I with lower *NF-κB2* mRNA levels had better 5-year survival outcome compared to patients with higher expression in univariate (Fig. 5d, P = 0.031), as well as in multivariate analysis using the age, grade, gender, histological subtype and primary location as coefficients (P = 0.028; HR, 0.043; 95% CI, 0.003–0.714).

In addition, patients without regional LN infiltration and lower *NF-κB2* mRNA levels had statistically significant improved survival rates after 5 years observation in comparison to thοse with higher expression (Fig. 5e, P = 0.004). Interestingly, the same correlation was also observed in multivariate analysis adjusted for age, grade, gender, histological subtype and primary location (P < 0.001; HR, 0.043; 95% CI, 0.007–0.245). On the contrary, patients with infiltrated N1 or N2 LNs and increased *NF-κB2* mRNA expression had better survival than patients with decreased *NF-κB2* gene expression. This association was statistically significant using Cox proportional hazards models adjusted for age, grade and primary location (P = 0.007; HR, 4.673; 95% CI, 1.519–14.372), but not in univariate analysis where it approached but did not quite achieve statistical significance (Fig. 5f, P = 0.059).

### Expression levels of NIK and RelB were correlated with relapse rate

**Figure 6:**
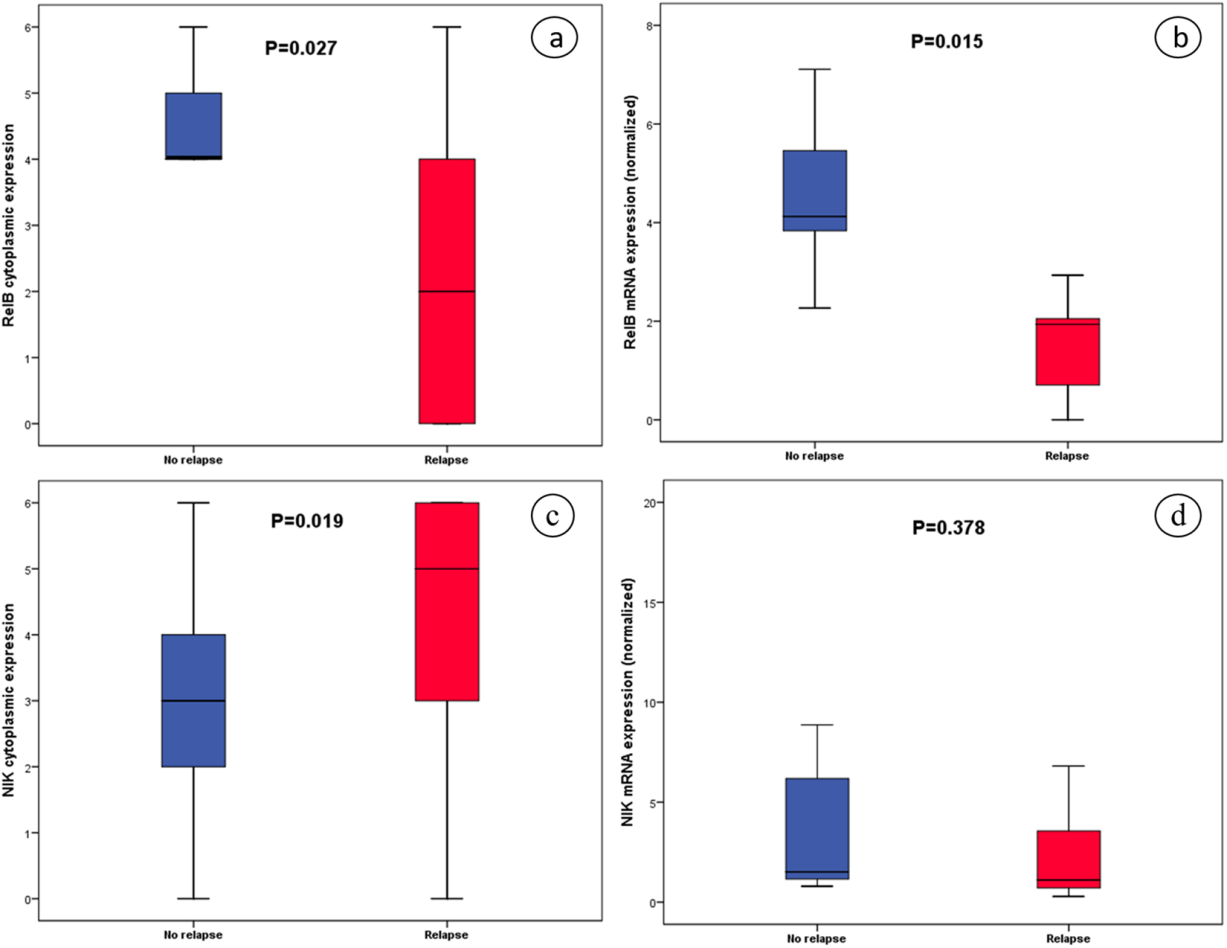
Boxplots of relapse status with regard to (**a**) RelB cytoplasmic, (**b**) RelB mRNA expression, (**c**) NIK cytoplasmic and (**d**) NIK mRNA expression.

Expression levels of RelB and NIK were associated with relapse rate (Fig. 6). In particular, RelB cytoplasmic signal was inversely associated with relapse rate, with higher cytoplasmic signal being related to lower relapse rate in univariate analysis (Fig. 6a, P = 0.027) as well in multivariate analysis (P = 0.029; HR, 0.496; 95% CI, 0.264–0.930). In concordance with this finding, higher *RelB* mRNA levels were also correlated with lower relapse rate (Fig. 6b, P = 0.015).

Association of relapse rate with NIK cytoplasmic expression was also observed, with lower expression being related to no relapse (Fig. 6c, P = 0.019). This association was also observed in logistic regression analysis using age, stage, maximum diameter, histological subtype and gender as coefficients (P = 0.028; HR, 2.87; 95% CI, 1.119–7.364). On the contrary, *NIK* mRNA levels weren’t correlated with relapse status (Fig. 6d) and, similarly, no significant association was found between Bcl3 and NF-κΒ2 expression, either in protein or in mRNA expression levels, with respect to relapse rate.

### NIK expression is related to regional lymph node infiltration

Cytoplasmic NIK expression in primary lesions was associated with infiltration of lymph nodes (Supplementary Fig. 1a,b, P = 0.039), while this association was not observed for mRNA expression (P = 0.743). Patients with infiltrated lymph nodes at baseline had lower cytoplasmic NIK expression compared to patients with absence of lymph node infiltration. On the contrary, no association was found between cytoplasmic NF-κΒ2 expression, RelB, Bcl3 and NF-κΒ2/ RelB “co-expression” and regional lymph node infiltration. Interestingly, NF-κΒ2/ Bcl3 “co-expression” was associated with lymph node infiltration, with concurrent signal for both molecules being higher in patients without lymph node disease (P = 0.014).

### Relations amongst the molecules

In addition, RelB cytoplasmic expression was correlated with RelB nuclear expression (P < 0.001) and Bcl3 cytoplasmic signal (P = 0.005), while the last was also associated with Bcl3 nuclear expression (P < 0.001). In addition, NIK cytoplasmic expression was related to Bcl3 cytoplasmic expression (P = 0.032) as well as to *NIK* mRNA levels (P = 0.017) and to *Bcl3* mRNA levels (P = 0.003). Additionally, Bcl3 nuclear expression was associated with *NF-κΒ2* mRNA levels (P = 0.027). *RelB* mRNA levels were associated with *NIK* mRNA (P = 0.008), *NF-κΒ2* mRNA (P < 0.001) and *Bcl3* mRNA levels (P < 0.001).

### Associations of studied molecules with pathological parameters

Interestingly, patients of stages I and IV were found to have lower NF-κΒ2 cytoplasmic expression compared to patients of stages II-III (P = 0.018). Similar differences were not detected in mRNA levels, as gene expression of *NF-κΒ2* was stable across stages (P = 0.356).

Furthermore, protein and/or mRNA levels were correlated with tumor size. In particular, higher levels of *NF-κΒ2* mRNA were related to larger primary lesions (P = 0.020). On the contrary, lower nuclear expression of Bcl3 was associated with larger maximum diameter of primary lesions (P = 0.039). In addition, using a two-tier grading system, tumors with high grade displayed higher expression of cytoplasmic NF-κΒ2 and NF-κΒ2 mRNA levels (Supplementary Fig. 2a, P = 0.046 and Supplementary Fig. 2b).

With regard to histological subtype, expression of RelB and NIK but not NF-κΒ2 and Bcl3 was statistically significant. RelB cytoplasmic expression was higher in squamous-cell carcinomas compared to adenocarcinomas (Supplementary Fig. 3a, P = 0.003). In addition, RelB nuclear expression was detected in higher levels in squamous carcinomas than adenocarcinomas (Supplementary Fig. 3b, P = 0.039). No difference was found in mRNA levels between adenocarcinomas and squamous cell carcinomas, while both had lower *RelB* mRNA levels compared to large cell carcinomas. In addition, cytoplasmic signal for NIK in squamous cell carcinomas was higher compared to adenocarcinomas (Supplementary Fig. 3c, P = 0.022), while no difference was found in mRNA levels.

## Discussion

The involvement of the alternative pathway of NF-κΒ has been increasingly recognized in lung cancer initiation, progression and clinical outcome as well as in response to treatment^17,20^. We have demonstrated previously that NF-κΒ2 and RelB are overexpressed in non-small-cell carcinomas^17^. Prompted by the previously reported preliminary data, we sought to clarify further the role of this pathway in NSCLC by assessing protein and gene expression of NF-κΒ2, RelB, Bcl3 and NIK in a bigger cohort. We showed that the expression of the major intracellular components of the NF-κΒ alternative pathway i.e. NF-κΒ2, RelB, NIK and Bcl3 are particularly deregulated in NSCLC. All four proteins as well as *RelB* mRNA levels were increased in neoplastic tissues compared to tumor-adjacent, non-cancerous tissues. In addition, expression levels of RelB, Bcl3 and NF-κΒ2 were associated with OS and those of NIK and RelB with relapse rate. Furthermore, NIK and NF-κΒ2 were related to regional lymph node infiltration. These findings further reinforce the evidence of the activation of this pathway in lung cancer, with findings to be consistent with previous research^17,18,21^.

Overexpression of NF-κΒ2 has been documented also in hematological cancers (cutaneous T lymphomas, NK/T lymphomas), as well as in a plethora of solid tumors such as myeloid thyroid cancer, pancreatic adenocarcinoma, breast, prostate, esophageal, NSCLC and colon cancers^17,22–30^. Moreover, the observed overexpression of NF-κΒ2 in NSCLC is consistent with the overexpression of the TNF receptor family member, LTβR (lymphotoxin-β receptor), that occurs in 87 to 96% of a wide range of solid tumors, including lung cancert^21,31^. LTβR is upstream of NF-κΒ2 and its activation results in the activation of the NF-κΒ alternative pathway^21^. In addition, another activator of the alternative NF-κΒ pathway, CD40 and its ligand, CD154, have been found to be overexpressed in 51.9 and 58.9% of NSCLC patients, respectively^32^. This further reinforces the fact that this pathway is undergoing deregulation.

Notably, one of the major findings of this study is the correlation between high cytoplasmic expression of RelB and poor OS in NSCLC patients. Our findings are corroborated by a number of other studies^33–35^. OS rates from the KM estimate, based on mRNA levels from publicly available databases, also suggest a significant difference for adenocarcinomas but not for squamous carcinomas (Supplementary Fig. 4). In addition, low RelB activity has been related to a favorable survival of patients with chronic lymphocytic leukemia (CLL)^33^. Furthermore, an association of high RelB protein levels with shorter OS of NSCLC patients has also been reported^34^. Recently, the prognostic significance of RelB levels has also been shown for patients with grade III and IV gliomas, whereby low RelB levels were associated with longer OS^35^.

A possible explanation for the translational value of RelB may lie on the functional connection of RelB with cancer cell growth, migration, and invasion. In DU145 prostate cancer cells, RelB seems to function as an oncogene^36^. Also, RelB in combination with RelA activity sustains the basal survival of CLL cells and renders them sensitive to proteasome inhibition^33^. In addition, RelB in advanced ovarian cancers, supports tumor-initiating cells through the cancer stem-like associated enzyme aldehyde dehydrogenase (ALDH), and the loss of RelB leads to reversion of chemoresistance and inhibition of tumorigenesis in mouse xenograft models^37^. RelB has also been implicated in fostering the stemness of osteosarcoma cells through the paracrine action of cancer-associated mesenchymal stromal cells^38^. Furthermore, in multiple myeloma, it is well known that RelB is able to exert a crucial anti-apoptotic role in malignant cells^39^. In addition, we have to note that RelB regulates gene expression by alternative mechanisms (e.g. epigenetic modifications, dimerization with dimers with the aryl hydrocarbon receptor) and it is not only part of the alternative signaling pathway of NF-kB^40,41^.

Similarly to RelB, elevated expression of Bcl3 is related to poor OS in stages I-II and to improved OS in stages III-IV patients. Elevated expression of Bcl3 at diagnosis has also been associated with poor prognosis of patients with multiple myeloma^42^. Similarly, an inverse correlation of Bcl3 expression with survival has been documented for patients with colorectal adenocarcinomas^43^. Importantly, this finding is consistent with our data of poor survival and in concordance with KM analysis especially with increased gene expression of Bcl3 in patients with stages I-II (Supplementary Fig. 5)^44^. On the contrary, increased Bcl3 expression was associated with improved OS in stage III and IV patients, a finding which is also consistent with the trend observed in survival analysis from KMplotter, although it didn’t reach statistical significance (Supplementary Fig. 5c). Notably, this stage-dependent association between mRNA levels and OS may reflect the different treatment regimens administered in the different stages of disease.

In addition to associations with OS, expression of members of the alternative NF-κΒ2 pathway was also related to local cancer relapse. Notably, our study is the first to our knowledge to report that RelB expression (in protein and mRNA level) was inversely associated with local relapse. Again, this observation is in agreement with the observations of others showing an inverse correlation of RelB expression with OS. Qin *et al*. showed that RelB is significantly related to distant metastasis in patients with NSCLC, indirectly supporting our findings. Moreover, it appears that this association may be tumor type specific, as no association was noted between expression of RelB and recurrence in ER-positive breast cancer patients^45^.

A linear association of relapse rate and regional lymph node infiltration with NIK cytoplasmic expression was also detected, with lower expression related to the absence of relapse. These findings may reflect NIK’s involvement in NSCLC progression through the activation of the NF-κΒ alternative pathway. Although, here, we document for the first time that NIK is overexpressed in NSCLC, it is well documented that NIK is overexpressed in culture and in *in vivo* models of NSCLC, representing a molecular switch of the activation of the NF-κΒ alternative pathway^46^. Furthermore, possible mechanisms through which NIK influences metastatic potential in lung cancer cells have been described. For example, NIK depletion induced apoptosis in A549 cells and in H1299 cells reduced their colony-forming efficiency, contributing to the oncogenic phenotypes of NSCLC cells^46^. In addition, NIK has been implicated in the regulation of breast cancer stem cells, while its inhibition can impair clonogenicity and tumorigenesis, not only through the activation of NF-κΒ, but also through the activation of the ERK1/2 pathway^47^.

Despite the promising results, we must acknowledge some limitations of our study. Tissue samples were all surgical specimens and therefore represent mainly early and locally advanced NSCLC patients, while stage IV is underrepresented. Additionally, a larger cohort could lead to even more robust associations especially in assessment of gene expression and in stratification analyses.

In conclusion, we report for the first time a comprehensive expression analysis of the major intracellular components of the alternative pathway of NF-κΒ and its prognostic significance in NSCLC. Our data provide strong evidence that this pathway is particularly deregulated in NSCLC, influencing concurrently the prognosis of patients.

## Methods

### Study design, population, tissue specimens and data collection

In this study, ethical guidelines of the Helsinki Declaration were followed (2013)^48^. Prior to study initiation, approval was granted by the Scientific Committee and the Committee on Research and Ethics of the University Hospital of Patras (Greece). Informed consent was obtained from all the participants.

**Figure 7:**
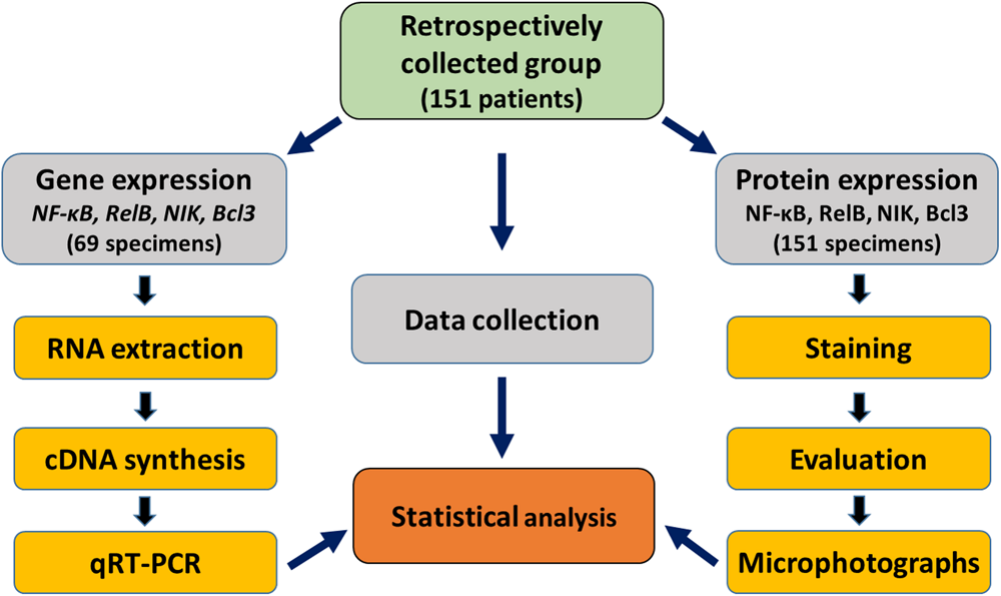
Overview of the workflow of the current study. Abbreviations: qRT-PCR, quantitative Reverse Transcription PCR.

Protein and mRNA expression of four members of the alternative pathway (NF-κB2, RelB, NIK and Bcl3) were studied in patients, who had undergone a curative resection of a lung tumor at the University Hospital of Patras between 2005 and 2010. This retrospective analysis was performed blindly using an archival database from the Pathology Department of the University Hospital of Patras which allowed access to formalin-fixed paraffin-embedded (FFPE) tissue specimens of both invasive NSCLC as well as adjacent non-neoplastic lung parenchyma. Clinical information was collected from medical records or through direct communication with the patients. Overall survival (OS) was defined up to a follow-up period of 60 months. The workflow of the study is described in Fig. 7.

### Immunohistochemical analysis

Immunohistochemistry was performed as described previously^17,18^. The primary antibodies used against NF-κΒ2, NIK and Bcl3 were mouse monoclonal, while the antibody against RelB was rabbit polyclonal. Conditions for each primary antibody (clonality, clone, dilution, antigen retrieval and incubation time) are presented in Supplementary Table S2. The Envision detection kit (DAKO) was used for detection and diaminobenzidine (DAB) was used as the chromogen for visualization according to the manufacturer’s instructions. Dehydrated Harris’ hematoxylin solution was used for counterstaining the sections. The specificity of the method was tested using protein blocking solution instead of the primary antibodies in consecutive sections. Inflammatory cells of the tumor microenvironment were used as internal positive controls.

### Evaluation of immunohistochemistry

All slides were assessed independently and blinded to each case by one pathologist (H.P.) and one investigator (F.D.). The histological type and tumor grade were confirmed based on the 2004 WHO classification^49^. Evaluation of the immunohistochemical signal was performed as described previously^17,18^. Cases were considered positive when staining was noted in >10% of cells. The distribution and intensity of the NF-κB2, RelB, NIK, and Bcl3 signals were used to estimate NF-κB2, RelB, NIK, Bcl3 expression. Staining was graded on a scale of 0–3 according to the intensity and the percentage of immunopositive cells as follows: 0: no staining or <10% positive cells; 1: weak staining in >10% of cells or moderate staining in 10–70% of cells; 2: moderate staining in >70% of cells or strong staining in 10–70% of cells; 3: strong staining in >70% of cells. NF-κB2, RelB, NIK, and Bcl3 protein expression in cancer cells was categorized in three groups (high vs medium vs low) using as a cut-off the 33^rd^ and 66^th^ percentiles or cut-offs derived from X-tile software^50,51^. For each slide, a total score was calculated as the sum of the intensity and the distribution (values ranging between 0 and 6). In order to obtain microphotographs, a Nikon DXM 1200 C digital camera with ACT-1C software mounted on a Nikon Eclipse 80i microscope (Nikon Instruments Inc., Melville, NY, USA) was used.

### Gene expression analysis by quantitative real-time PCR analysis (qRT-PCR)

#### RNA preparation

Four 10 μm slides of neoplastic, and when available, paired, non-malignant, adjacent FFPE tissue specimens from 69 NSCLC patients were used to extract RNA samples using the commercially available kit, NucleoSpin® totalRNA FFPE Kit (MACHEREY-NAGEL, GmbH & Co., Düren Germany), according to the manufacturer’s instructions. Isolated RNA samples were then treated with DNase (Ambion, Austin, TX, USA), and total RNA was quantified using a Nanodrop-1000 spectrophotometer (NanoDrop, Fisher Thermo, Wilmington, DE, USA) and stored at −80 °C.

#### cDNA synthesis

A total of 3 μg of RNA was reverse transcribed into cDNA using 100U of Superscript III Reverse Transcriptase (Life Technologies), 300 ng of random nonamer primers (Foundation for Research and Technology-Hellas, Crete, Greece) and 100 nM dNTPs (Stratagene) in a total volume of 50 μl. To control the RNA samples for DNA contamination, a no enzyme control was used. Additionally, a commercially available RNA sample (Stratagene) was used as a calibrator in every batch of reverse transcription reactions to account for run to run variablility. The mixture was incubated in a C1000 Touch thermal cycler (Bio-Rad) at 25 °C for 5 minutes, 50 °C for 60 minutes, and 70 °C for 15 minutes. CDNA was diluted to 15 ng/μl and stored at −20 °C.

#### Quantification of gene expression

Expression levels of the *NF-κB2*, *RelB*, *NIK* and *Bcl3* genes were quantified by real-time PCR (qPCR) assays. Specific primers and probes for *NF-κB2*, *RelB*, *NIK*, *Bcl3* and *IPO8* genes (Importin 8 was used as a reference gene) were designed to bind to all isoforms using OligoAnalyzer 3.1 (Integrated DNA Technologies, Inc.)^52^, according to the sequences provided in NCBI (http://www.ncbi.nlm.nih.gov/). Primers and probes were synthesized by IDT. Primer sequences and reaction conditions can be provided upon request. The qPCR reactions were carried out in triplicates, in a total volume of 20 μl, containing 5 μl of cDNA in 1× Kapa Probe Fast Master Mix (KAPA BIOSYSTEMS, Woburn, MA, USA) in an MX3000p cycler (Stratagene, La Jolla, CA, USA). Relative expression levels were calculated using the LinReg Program^53^ and were normalized to levels obtained for the calibrator sample and to *IPO8* levels.

### Statistical analysis

The Statistical Package for Social Sciences Version 17 (SPSS, Chicago, IL, USA) was used for statistical analysis. Associations between protein expression and clinicopathological parameters of patients were assessed by using the χ^2^ test for nominal variables and the Kruskal-Wallis or the Mann-Whitney tests for ordinal variables. T test was used for continuous variables such as gene expression. Spearman’s correlations were used to assess associations between variables. The association of expression levels with relapse rates was evaluated using logistic regression models, adjusted for coefficients. The Kaplan-Meier method and the log rank test were used for the estimation of survival rates. The prognostic significance of the studied molecules was evaluated by Cox regression analysis. The X-tile software was used in order to provide the best cut-off points^51^. For all comparisons, statistical significance was defined as P < 0.05.

## Supplementary information

Supplementary_Figure_1

Supplementary_Figure_2

Supplementary_Figure_3

Supplementary_Figure_4

Supplementary_Figure_5

Supplementary Table S1

Supplementary Table S2
