# Supplementary material for: Expression Of Intracellular Components of the NF-κB Alternative Pathway (NF-κB2, RelB, NIK and Bcl3) is Associated With Clinical Outcome of NSCLC Patients
Source: Sci Rep. 2019 Oct 4;9:14299. doi: 10.1038/s41598-019-50528-y (PMC6778110; doi:10.1038/s41598-019-50528-y)
Supplement: Supplementary file 6 — Supplementary Table S1 [file 41598_2019_50528_MOESM6_ESM.pdf]

Expression Of Major Intracellular Components of the NF-κB Alternative Pathway (NF-κB2, RelB, NIK and Bcl3) is Associated With Clinical Outcome of NSCLC Patients

[1§]Foteinos-Ioannis D. Dimitrakopoulos, [1§]Anna G. Antonacopoulou, [1§]Anastasia E. Kottorou, [2]Nikolaos Panagopoulos, [1]Fotini Kalofonou, [3]Fotios Sampsonas, [4]Chrisoula Scopa, [5]Melpomeni Kalofonou, [1]Angelos Koutras, [1]Thomas Makatsoris, [2]Dimitrios Dougenis, [6]Helen Papadaki, [7]Malcolm Brock, [1*]Haralabos P. Kalofonos

[1]Molecular Oncology Laboratory, Division of Oncology, Department of Internal Medicine, Medical School, University of Patras, Patras, Greece.

[2] Department of Cardiothoracic Surgery, Medical School, University of Patras, Patras, Greece.

[3] Department of Respiratory Medicine, University Hospital of Patras, Patras, Greece.

[4]Department of Pathology, Medical School, University of Patras, Patras, Greece.

[5]Institute of Biomedical Engineering, Imperial College London, London, United Kingdom.

[6]Department of Anatomy, Medical School, University of Patras, Patras, Greece.

[7]Division of Thoracic Surgery, Department of Surgery, School of Medicine, Johns Hopkins University, Baltimore, MD, USA.

§ These authors contributed equally to this work.

*Corresponding author: Prof. Haralabos P. Kalofonos, Division of Oncology, Department of Internal Medicine, Medical School, University of Patras, Rion-Patras 26504, Greece. Tel.: +30 2610999535, Fax: +30 2610994645, E-mail: kalofonos@upatras.gr

Clinicopathological characteristics	Cases<br><br>*n* (%)
Total	151 (100)
Age (years) Median (range)	66 (40-84)
Gender	
Total	151 (100)
Male	139 (92.1)
Female	12 (7.9)
Smoking (pack-years)	
Total	151 (100)
Cases (%)	59 (39.1)
Mean (range)	87.63 (20-165)
NA	92 (60.9)
Primary location	
Total	151 (100)
Left lung	66 (43.7)
Right lung	85 (56.3)
NA	-
Histology	
Total	151 (100)
Squamous	86 (57.0)
Adenocarcinoma	54 (35.8)
Large carcinoma	10 (6.6)
NA	1 (0.6)
Stage	
Total	151 (100)
I	59 (39.1)
II	44 (29.1)
III	45 (29.8)
IV	2 (1.3)
NA	1 (0.7)
Grade	

**Total**	**151 (100)**
**I**	**4 (2.6)**
**II**	**70 (46.4)**
**III**	**67 (44.4)**
**NA**	**10 (6.6)**
**Maximum diameter (cm)**	
**Total**	**151 (100)**
**Cases (%)**	**147 (97.4)**
**Mean (range)**	**5.09 (0.70-21.00)**
**NA**	**4 (2.6)**
**Lymph node infiltration**	
**Total**	**151 (100)**
**No**	**79 (52.3)**
**Yes**	**65 (43.0)**
**NA**	**7 (4.6)**
**Metastasis (adrenals)***	
**Total**	**151 (100)**
**No**	**29 (19.2)**
**Yes**	**7 (4.6)**
**NA**	**115 (76.2)**
**Metastasis (liver)***	
**Total**	**151 (100)**
**No**	**30 (19.9)**
**Yes**	**5 (3.3)**
**NA**	**116 (76.8)**
**Metastasis (brain)***	
**Total**	**151 (100)**
**No**	**30 (19.70)**
**Yes**	**9 (6.0)**
**NA**	**112 (74.2)**
**Metastasis (bone)***	
**Total**	**151 (100)**
**No**	**23 (15.2)**

**Yes**	**17 (11.3)**
**NA**	**111 (73.5)**
**Metastasis (adrenals-liver-brain-bone)***	
**Total**	**151 (100)**
**No**	**9 (6.0)**
**Yes**	**36 (23.8)**
**NA**	**106 (70.2)**
**Survival (2 years)**	
**Total**	**151 (100)**
**Dead**	**56 (37.1)**
**Alive**	**93 (61.6)**
**NA**	**2 (1.3)**
**Survival (3 years)**	
**Total**	**151 (100)**
**Dead**	**75 (49.7)**
**Alive**	**71 (47.0)**
**NA**	**5 (3.3)**
**Survival (5 years)**	
**Total**	**151 (100)**
**Dead**	**91 (60.3)**
**Alive**	**55 (36.4)**
**NA**	**5 (3.3)**
**Relapse**	
**Total**	**151 (100)**
**No**	**14 (9.3)**
**Yes**	**23 (15.2)**
**NA**	**114 (75.5)**

Supplementary Table S1. Clinicopathological characteristics and survival data of NSCLC patients. Abbreviations: NA, data not available or unknown. * Metastasis status refers to metastases, which were developed the patients during the observation period.
