# Supplementary material for: Expression Of Intracellular Components of the NF-κB Alternative Pathway (NF-κB2, RelB, NIK and Bcl3) is Associated With Clinical Outcome of NSCLC Patients
Source: Sci Rep. 2019 Oct 4;9:14299. doi: 10.1038/s41598-019-50528-y (PMC6778110; doi:10.1038/s41598-019-50528-y)
Supplement: Supplementary file 7 — Supplementary Table S2 [file 41598_2019_50528_MOESM7_ESM.pdf]

Expression Of Major Intracellular Components of the NF-κB Alternative Pathway (NF-κB2, RelB, NIK and Bcl3) is Associated With Clinical Outcome of NSCLC Patients

[1§]Foteinos-Ioannis D. Dimitrakopoulos, [1§]Anna G. Antonacopoulou, [1§]Anastasia E. Kottorou, [2]Nikolaos Panagopoulos, [1]Fotini Kalofonou, [3]Fotios Sampsonas, [4]Chrisoula Scopa, [5]Melpomeni Kalofonou, [1]Angelos Koutras, [1]Thomas Makatsoris, [2]Dimitrios Dougenis, [6]Helen Papadaki, [7]Malcolm Brock, [1*]Haralabos P. Kalofonos

[1]Molecular Oncology Laboratory, Division of Oncology, Department of Internal Medicine, Medical School, University of Patras, Patras, Greece.

[2] Department of Cardiothoracic Surgery, Medical School, University of Patras, Patras, Greece.

[3] Department of Respiratory Medicine, University Hospital of Patras, Patras, Greece.

[4]Department of Pathology, Medical School, University of Patras, Patras, Greece.

[5]Institute of Biomedical Engineering, Imperial College London, London, United Kingdom.

[6]Department of Anatomy, Medical School, University of Patras, Patras, Greece.

[7]Division of Thoracic Surgery, Department of Surgery, School of Medicine, Johns Hopkins University, Baltimore, MD, USA.

§ These authors contributed equally to this work.

*Corresponding author: Prof. Haralabos P. Kalofonos, Division of Oncology, Department of Internal Medicine, Medical School, University of Patras, Rion-Patras 26504, Greece. Tel.: +30 2610999535, Fax: +30 2610994645, E-mail: kalofonos@upatras.gr

Antibody	Clonality	Company	Catalogue number	Clone	Dilution	Antigen retrieval conditions	Incubation time
NF-kB2	M	Santa Cruz	sc-7386	C-5	1:500	8mM sodium citrate, PH 6.0	2 hours (room temperature)
RelB	P	Santa Cruz	sc-226	C-19	1:500	8mM sodium citrate, PH 6.0	2 hours (room temperature)
NIK	M	Santa Cruz	sc-8417	A-12	1:20	1,2 mM EDTA,PH 8.0	2 hours (room temperature)
Bcl3	M	Novocastra	NCL-BCL3	1E8	1:40	1,2 mM EDTA, PH 8.0	2 hours (room temperature)

Supplementary Table S2. Primary antibodies and their clonality, clone, dilution, antigen retrieval and incubation time information. M: monoclonal, P: polyclonal.
